# 0295. Induction and repression effects of heat shock (HS) and LPS and modulatory effects of glutamine on blood mononuclear cells -hsprotein-72 from icu patients with severe sepsis, trauma and healthy controls

**DOI:** 10.1186/2197-425X-2-S1-P17

**Published:** 2014-09-26

**Authors:** E Briassouli, M Tzanoudaki, G Daikos, K Vardas, M Kanariou, C Routsi, S Nanas, G Briassoulis

**Affiliations:** 1st Department of Propaedeutic InternalMedicine, University of Athens, Athens, Greece; Department of Immunology - Histocompatibility, Specialized Center & Referral Center for Primary Immunodeficiencies - Paediatric Immunology, “Aghia Sophia” Children's Hospital, Athens, Greece; First Critical Care Department, University of Athens, Evangelismos Hospital, Athens, Greece; PICU, University of Crete, University Hospital, Heraklion, Greece

## Introduction

In severe sepsis (SS) or trauma-related systemic inflammatory response syndrome (SIRS), induction of heat-shock-protein-72 (HSP72) may protect cells from stress.

## Objectives

We compared the heat-shock (HS) with the lipopolysaccharide (LPS) induction/repression effect on HSP72 of peripheral blood mononuclear cells (PBMCs) in SS or SIRS patients and healthy-controls (H) and investigated any possible modulating glutamine (Gln) effect.

## Methods

PBMCs from 16/H, 11/SS, and 7/SIRS were incubated with 1µg/ml LPS or 43^o^ HS vs. no stimulation for 4h. In each group 3 experiments involved L-Ala-Gln10mM incubation 1h before (Gln-b) or after (Gln-a) induction, or no glutamine (1088 measurements). Intracellular Mean Fluorescence Intensity (MFI) levels of monocytes (mHSP72) or lymphocytes (lHSP72) were determined using Flow Cytometry.

## Results

In H-PBMCs, LPS did not affect mHSP72 (79±10 MFI vs. 78±13) or lHSP72 (7±1.7 vs. 7±2). HS induced mHSP72 (454±60, p< 0.0001) and lHSP72 (41±7, p< 0.0001) with or without Gln (p< 0.0001). Basal mHSP72 was higher in SIRS compared to H (144±25 vs. 78±10, p< 0.03). A HS-induction effect on SIRS-mHSP72 (394±108, p< 0.04) and lHSP72 (37±5, p< 0.02) was further enhanced by Gln-b (495±114, p< 0.01 and 58±14, p< 0.04). LPS suppressed SIRS-mHSP72 (120±54 vs. 144±25, p< 0.02) especially in the Gln-b group (107±19, p< 0.02). Basal Gln-b mHSP72 in SS was higher compared to H (112±16 vs. 69±10, p< 0.03). In SS-PBMCs HS, but not LPS, induced mHSP72 (492±56 vs. 108±19, p< 0.003). LPS repressed the SS-lHSP72 (10±2 vs. 17±2, p< 0.007) an effect attenuated by Gln-b (13±5).

## Conclusions

Heat shock greatly induces mHSP72 and lHSP72 of ICU patients' PBMCs. LPS may repress lHSP72 in septic or trauma patients. Glutamine pre-treatment may either enhance HS-induction or LPS-repression on mHSP72 or attenuate LPS-repression on lHSP72 in SS and SIRS groups.

